# Healthy at Home for COPD: An Integrated Digital Monitoring, Treatment, and Pulmonary Rehabilitation Intervention

**DOI:** 10.21203/rs.3.rs-5084150/v1

**Published:** 2024-11-15

**Authors:** Laurel O’Connor, Stephanie Behar, Seanan Tarrant, Pamela Stamegna, Caitlin Pretz, Jeanne Shirshac, Thomas Scornavacca2, Tracey Wilkie, Kimberly Fisher, Emil Tigas, Marie Mullen, Michael Hyder, Steven Wong, Brendon Savage, shaun Toomey, Biqi Wang, Adrian Zai, Eric Alper, Peter Lindenauer, Eric Dickson, John Broach, David McManus, Vik Kheterpal, Apurv Soni

**Affiliations:** University of Massachusetts Chan Medical School; University of Massachusetts Chan Medical School; University of Massachusetts Chan Medical School; University of Massachusetts Chan Medical School; University of Massachusetts Chan Medical School; UMass Memorial Medical Center; University of Massachusetts Chan Medical School; UMass Memorial Medical Center; University of Massachusetts Chan Medical School; University of Massachusetts Chan Medical School; University of Massachusetts Chan Medical School; University of Massachusetts Chan Medical School; University of Massachusetts Chan Medical School; CareEvolution (United States); University of Massachusetts Chan Medical School; University of Massachusetts Chan Medical School; University of Massachusetts Chan Medical School; University of Massachusetts Chan Medical School; University of Massachusetts Chan Medical School-Baystate Campus; University of Massachusetts Chan Medical School; University of Massachusetts Chan Medical School; University of Massachusetts Chan Medical School; CareEvolution (United States); University of Massachusetts Chan Medical School

## Abstract

**Background:**

Chronic Obstructive Pulmonary Disease (COPD) is a leading cause of morbidity and mortality in the United States. Frequent exacerbations result in higher use of emergency services and hospitalizations, leading to poor patient outcomes and high costs.

**Objective:**

Demonstrate the feasibility of a multimodal, digitally enhanced remote monitoring, treatment, and tele-pulmonary rehabilitation intervention among patients with COPD.

**Methods:**

In this pilot clinical trial, community-dwelling adults with moderate-severe COPD were enrolled in a multimodal digital monitoring and treatment program including a Fitbit wearable device, study app that tracked COPD-related symptoms, on-demand mobile integrated health (MIH) services for acute home-based treatment, and tele-pulmonary-rehabilitation. Participants were enrolled in the program for six months. COPD severity and health-related quality of life assessments were performed at baseline, 3 months, and 6 months. Primary feasibility outcomes include recruitment and retention rate, and participant protocol fidelity, which were reported descriptively. Exploratory clinical outcomes included patient-reported quality of life, activation, and intervention satisfaction.

**Results:**

Over 18 months, 1,333 patients were approached and 100 (7.5%) were enrolled (mean age 66, 52% female). Ninety-six participants (96%) remained in the study for the full enrollment period. Fifty-five (55%) participated in tele-pulmonary-rehabilitation. Participants wore the Fitbit for a median of 114 days (IQR 183.6) and 16.85 hours/day (4.05), resulting in a median of 1133 minutes (243) per day. Completion rates for scheduled instruments ranged from 78–93%. Nearly all participants (85%) performed COPD ecological momentary assessment at least once with a median of 4.85 recordings. On average, a 2.48-point improvement (p=0.03) in COPD Assessment Test Score was observed from baseline to study completion. The adherence and symptom improvement metrics were not associated with baseline patient activation measures.

**Conclusions:**

A multimodal intervention combining preventative care, symptom and biometric monitoring, and home treatment was feasible in adults living with COPD. Participants demonstrated high protocol fidelity and engagement and reported improved quality of life.

## Introduction

Chronic obstructive pulmonary disease (COPD) is a major public health burden responsible for 150,000 deaths, 873,000 emergency department visits, and 700,000 hospitalizations annually in the United States. It costs $50 billion to treat every year: 70% of these expenditures are for acute care services. (1–5)Unplanned use of emergency services represents critical events in the healthcare trajectories of patients with COPD, and it contributes to inconsistencies in treatment, patient distress, and further acute care needs.(2, 6–9) Between 30–50% of patients with COPD experience at least one acute exacerbation a year, and a history of severe COPD exacerbations is a risk factor for future emergency service needs: one in five patients hospitalized for COPD exacerbation is re-admitted within 30 days.(9) Strategies are needed to decrease the incidence of severe COPD exacerbation to improve disease management, reduce costs, and support patient quality of life.

Early detection of clinical signs of COPD exacerbations paired with coordinated therapeutic interventions, including pharmacotherapy, close clinical monitoring, and pulmonary rehabilitation, can prevent severe clinical deterioration, acute care needs, and future morbidity.(10–14) (15) Implementing such interventions before hospital admission for COPD is more effective than initiating interventions during or after hospitalization to prevent future hospitalization and mortality.(15) Therefore, the best opportunity to prevent COPD-associated hospitalization and recurrent episodes is before an index hospitalization. However, detecting clinical signs of COPD exacerbation in community-dwelling adults expeditiously and initiating a timely clinical response remains challenging.

Investigative community-based interventions targeting COPD management have largely focused on preventative and post-acute care with relatively few projects examining the management of acute symptoms. Pilot studies of home monitoring systems using patient-reported changes in symptoms and wearable sensors that transmit biometric data show promise for accurately predicting clinical deterioration.^20,21^ Additionally, pulmonology rehabilitation and lifestyle coaching have been shown to improve clinical outcomes in COPD including quality of life, functional capacity, and patient-reported dyspnea, which in turn decreases acute care needs.(16–18) Virtual pulmonary coaching and rehabilitation in particular may be effective because it mitigates obstacles to healthcare access and therefore facilitates superior patient compliance.(16, 19) However, to be maximally effective, pairing preventative care and clinical monitoring with a structured means to deliver timely acute care is necessary to ensure that detected clinical deterioration can be addressed and mitigated. Thus, a proactive, multidisciplinary infrastructure is needed to review the alerts and respond to them based on home monitoring to prevent added burden for the patients and ensure early initiation of treatment.

One viable solution to the limitations of remote monitoring may be Mobile Integrated Health (MIH) models, which are healthcare delivery initiatives that leverage mobile resources, including specially trained paramedic-level clinicians, to care for patients at home. Equipped with mobile diagnostic equipment and a portable medication formulary, highly trained MIH paramedics are dispatched into the community on-demand to perform in-home medical evaluations and treatment(s) in consultation with an actively involved supervising physician. MIH programs may respond at the request of patients or their caregivers when acute symptoms or ominous changes in biometric signals are detected.(20–22) MIH programs often offer 24/7 availability and, unlike traditional emergency services, are designed to facilitate treatment in the community with transportation to the hospital only for patients too unstable to be managed at home. By providing evaluation and treatment at home, MIH programs are designed to expand the reach and longitudinal capabilities of patients’ ambulatory providers. Pilot studies have shown that these programs are safe and can result in decreased emergency services utilization and claims costs, and improved patient satisfaction for a variety of health conditions.(21, 23–25)

A multidisciplinary strategy incorporating remote pulmonary rehabilitation and biometric monitoring, with aggressive mobile treatment during acute episodes may overcome many of the barriers that prevent timely recognition of acute COPD exacerbation and keep patients from accessing timely care. Digital and field-based interventions eliminate obstacles such as lack of transportation, scheduling difficulties, hesitancy to present to healthcare facilities, and low health literacy.(26) Furthermore, biometric monitors and in-home visits from clinicians provide valuable information about patients’ disease states in their lived environment, offering a more complete illustration of their health status and facilitating more informed management than telehealth alone. Few interventions examine the feasibility or impact of a unified multifaceted model that integrates these valuable resources into a single, integrated approach to community-based care for COPD. The objective of this study is to describe the feasibility of a multidisciplinary digital and community-based intervention for patients living with COPD to improve access to preventative and acute care for acute COPD exacerbation.

## Methods

### Setting and Participants

This decentralized clinical study was conducted through an academic tertiary care center. A detailed explanation of the study protocol is described elsewhere.(27) Inclusion criteria for participation included receiving healthcare through the affiliate hospital system, being 18 years of age or older at the time of recruitment, English speaking, and having a diagnosis of COPD. Subjects were also required to have access to a smartphone (with iOS or Android) to download and use the study apps and live within the regional geographic area served by the system’s MIH program, which included nine adjacent cities and towns. Patients who could not consent, did not understand English, did not have internet access on their smartphone while at home, were enrolled in another investigational clinical trial at the time of recruitment, or had ever been enrolled in any Wellinks pulmonary support program (with whom we partnered with for the full-intervention arm) were excluded. The study was approved by the WIRB-Copernicus Group Institutional Review Board and is registered at Clinicaltrials.gov (NCT06000696).

### Recruitment and enrollment

Initial screening for eligibility was performed via a query of the hospital system’s electronic health record (EHR) for patients who had a diagnosis of COPD and carried a moderate (25–50%) predicted risk of admission for COPD exacerbation within six months. Admission risk was ascertained by a predetermined risk stratification protocol derived from the number of acute care episodes (ED visits and hospitalization) and the number of COPD-related medication changes in the two years before study enrollment as proxy variables for COPD severity. We refined our recruitment to those patients that were between the second and fourth quintiles of the count variable such that our cohort was comprised of patients with moderate severity COPD.

Participants who were interested in participating after being approached were prompted to undergo further screening for eligibility and if eligible, enrollment and consent procedures via a customized study app on a platform called MyDataHelps, an application hosted by CareEvolution. Research coordinators were available to support subject enrollment via video or audio call for any subject that requested support.

Once participants completed consenting procedures, a “welcome kit” was shipped to their residence containing all necessary study-related materials including a Fitbit Charge 5 smartwatch, additional literature about the study and the affiliated MIH program, and information regarding how to request an MIH visit on-demand. Subjects were remotely guided through setting up and using the smartwatch, educated on the use of the study app, and provided any additional support needed to initiate participation in the study remotely by the study team. Once onboarded, participants were asked to partake in study procedures for six months.

### Study Procedure

The Healthy at Home intervention was comprised of several complementary components providing biometric monitoring, symptom tracking, mobile acute care services through the MIH program, and optional digital pulmonary rehabilitation. Table 1 summarizes each component and its interaction with other study constituents.

#### Remote patient activity monitoring

Participants were asked to wear the smartwatch daily, including at night, to collect data including daily steps, heart rate, oxygen saturation, and sleep patterns. This information was visible to study coordinators and investigators, as well as the MIH paramedics, through the MyDataHelps platform. For participants who opted into the full-intervention arm, this information was also shared with the tele-pulmonary-rehabilitation care team through the MyDataHelps platform.

#### Patient Surveys

All study participants were asked to complete a series of instruments throughout the course of the six-month study period through the MyDataHelps app. Participants were notified of outstanding surveys and were prompted to complete them through app push notifications according to the preset study schedule. In addition to demographic questions asked at baseline, participants were prompted to complete NIH-PROMIS COPD questionnaires, the Patient Activation Measure (PAM), and the Modified Medical Research Council Dyspnea Scale at enrollment, 3 months, and 6 months.(28–30) Participants were also prompted to complete a COPD self-assessment test monthly and a single-item wellness measure weekly. Finally, CLEAR-Sx, Ex, and Rx surveys were triggered ad-hoc by participants’ biometric data based on set metrics or could be completed as desired by the participant.(31) The chosen biometric alerts are summarized in Supplementary Table 2. In addition to their self-reported surveys, all subjects were asked for permission to link their electronic health records to the study app so that claims and healthcare utilization patterns could be tracked throughout their participation.

#### Virtual Comprehensive Pulmonary Support Service

All participants were given the option to enroll in an additional portion of the study providing them access to a virtual pulmonary care program that provides support for COPD patients, through a commercially available service offered by Wellinks Inc. This service includes live coaching to support patient education, treatment adherence counseling, and goal setting, as well as a virtual pulmonary rehabilitation program that delivers a home-based exercise plan including safety instructions and COPD-specific breathing techniques.

#### MIH Integration

On-demand, field-based clinical support was offered to all study participants, through the institution’s affiliate mobile integrated health (MIH) program for the duration of their participation.^24,25^ Participants, their caregivers, and the tele-pulmonary-rehabilitation coaching team were empowered to request an MIH visit for acute clinical symptoms (such as worsening shortness of breath) by calling the MIH clinical hotline. The community paramedics team is available 24 hours a day, 7 days a week, and presents to patients’ homes within 2 hours of a request. Paramedics evaluate and treat patients aided by mobile diagnostic tools and medications as well as live telehealth support from an on-call supervising physician. The MIH program is specifically equipped to initiate treatment for COPD exacerbation with inhaled bronchodilators and parenteral steroids and antibiotics. If a patient was too acutely ill to remain at home, the patient was diverted into the emergency services system.

To unify the study components, and streamline care, the community paramedic team and their supervising physicians had access to participants’ study dashboards so that they could review their aggregated clinical data and monitor any changes in participants’ biometric patterns. The study app also contained a “Call MIH” button which enabled participants to easily connect with the MIH hotline for an in-home assessment. Clinical care provided by the MIH paramedics for the study’s participants was identical in practice to the care provided outside the study.

### Measures and Analysis

The primary objective of this pilot study was to assess the feasibility of the Healthy at Home program. Primary feasibility measures included study recruitment and retention rate. Secondary outcomes included participant fidelity to study instruments, adherence to sensor use, use of the MIH program, and adoption rate of virtual pulmonary support and coaching activities. Exploratory clinical outcomes included COPD impact on patient quality of life, measured by the COPD assessment test (CAT) score, NIH PROMIS for COPD, and modified Medical Research Council Dyspnea Scale (mmRC). (28–30) These patient-facing instruments were reported descriptively and compared between baseline, three, and 6 months of the study. Lastly, operational feasibility measures included the number of study-related triggers that resulted in ambulatory encounters, MIH encounters, and emergency services use, MIH visit escalation rate, and acute-care visits not prompted by study triggers.

The sample size was chosen to assess the feasibility of recruitment and fidelity to the intervention aligning with the standards for feasibility studies; no *a priori* sample size was calculated. ^27, 28^ Findings were conveyed in aggregate and also stratified across patient activation categories as measured by baseline participant-reported PAM scores in order to describe findings across levels of participants’ baseline level of activation in their health. For PAM-stratified results, participants were classified as 1) PAM Category 1 or 2: Disengaged and Overwhelmed or Becoming Aware but Still Struggling [Score 0–55.1] 2) PAM Category 3: Taking Action [score 55.2–67] and PAM Category 4: Maintaining Behaviors and Pushing Further [score 67.1–100]. ANOVA testing was used to evaluate the significance of differences between baseline and follow up instrument scores. All statistical analysis was completed using STATA (V 17.0) (StataCorp, College Station, TX).

## Results

### Study Participants

**Figure 1:**
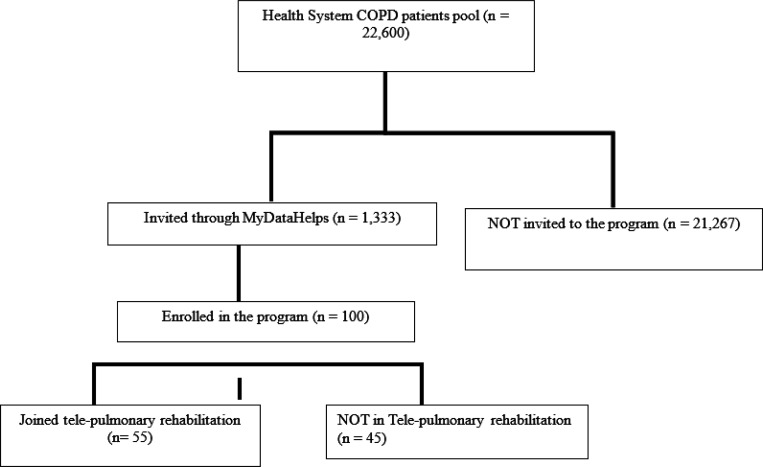
Healthy at Home CONSORT Diagram

Participant recruitment was completed over eighteen months; Figure 1 depicts the participant recruitment CONSORT diagram. The overall recruitment rate was 7.5%. In total 100 subjects were enrolled (mean age 66, 52% female). Table 2 summarizes participant demographic characteristics. Two patients withdrew voluntarily before study completion. Additionally, two patients died while enrolled of causes unrelated to the study, resulting in a retention rate of 98% and an overall completion rate of 96%. The study participants were predominantly white (n =83) and non-Hispanic/Latino. Supplemental Table 1 depicts the demographical distribution of the overall population from which subjects were drawn, broken down by group into patients who were and were not invited, and again by those who did and did not respond. Compared to the overall population, enrolled participants had a lower mean age, but the distribution of sex and race were similar. Of the 100 enrolled subjects, 90 completed the baseline PAM instrument. Nine had very low/low baseline PAM scores (Category 1–2: Disengaged and Overwhelmed or Becoming Aware but Still Struggling [Score 0–55.1]); 30 had Pam Category 3 scores (Taking Action [score 55.2–67]) and 31 had PAM Category 4 scores (Maintaining Behaviors and Pushing Further [score 67.1–100]).

### Participant Engagement

**Figure 2:**
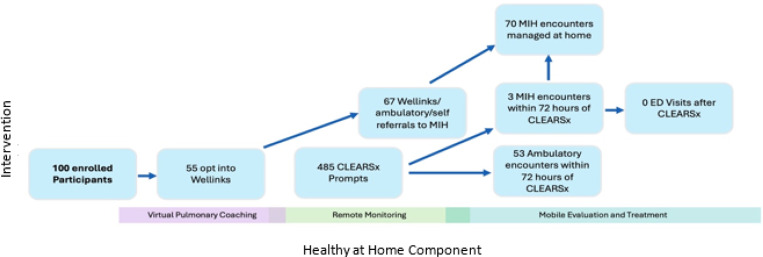
Participant Engagement: Participants interacted with Pulmonary Rehabilitation, MIH and remote monitoring throughout the study period

Table 3 summarizes participant engagement by intervention subtype. More than half (n=55, 55%) of participants opted into tele-pulmonary-rehabilitation and coaching. Participants wore the Fitbit for a median of 114 days during enrollment, with a median daily use of 16.85 hours which yielded a median of 1133 minutes during which their heart rates were detectable. Survey instrument completion rates varied between 78–93% for scheduled instruments, with baseline surveys having the highest response rate, and repeated instruments yielding lower participant compliance. Eighty-five discrete participants completed a total of 485 CLEAR-Sx ad-hoc surveys that were activated proactively by the participant, generated in response to biometric triggers, or were prompted by random nudges programmed into the study app. In 53 instances, subjects had a subsequent ambulatory encounter related to COPD within 72 hours of the CLEAR-Sx survey and in 3 instances, participants had an MIH visit; all three were treated at home. There were no ED visits within 72 hours of a CLEAR-Sx survey. One subject had a COPD-related ED visit with no preceding CLEAR-Sx survey. In an additional 67 instances, participants self-referred themselves or had an ambulatory clinician refer them for an MIH visit without a preceding CLEAR-Sx survey. Figure 2 depicts the flow of intervention activities and resultant healthcare utilization patterns.

### Intervention Effect Measures

#### Patient Quality of Life

Table 4 summarizes patient quality of life ratings throughout the study period, including the CAT, NIH COPD PROMIS, MMRC, and Patient Activation Measure. The mean participant CAT score decreased by an average of 2.48 points (p=0.03) between baseline and completion of the study.

## Discussion

This study demonstrates the feasibility of a novel approach to COPD management utilizing an intervention that provides community-based care by integrating digital and mobile tools to detect and treat COPD exacerbation. Preliminary evidence also suggests the program improved patient quality of life and decreased COPD-related distress. This approach-using integrated digital and mobile tools to support patients with COPD, particularly during acute events-warrants further investigation to determine if it provides a viable contributory solution to the significant burden that COPD imposes on individuals, health systems, and communities.(32) COPD is particularly challenging to patients and hospitals during time-sensitive, acute exacerbation events where the current mainstay of acute COPD care is based in brick-and-mortar clinical settings. This presents considerable accessibility concerns that may be overcome by remote and digital solutions. (31–34) Our work seeks to expand on innovation in self-management strategies and remote monitoring by unifying remote and digital treatment to create a more comprehensive community-based healthcare encounter for COPD management.

In the present study, 100 subjects were successfully recruited over eighteen months. The intervention demonstrated a high retention rate: 96% of subjects successfully remained in the study for the full six-month enrollment period. Subjects exhibited high fidelity to the study protocol including correctly and frequently using the wearable monitors, completing study instruments, and engaging with MIH for acute symptom management. These findings suggest that despite the multi-component intervention’s complexity, patients could engage effectively with its multiple elements. These findings remained consistent even when stratified across levels of baseline patient activation, an important driver of self-management and outcomes in COPD care.(35, 36) Given that low patient activation is a common challenge in COPD management and is associated with poor outcomes, it is crucial to ensure that an intervention, particularly a complex one, aimed at managing COPD is adopted even by participants with lower baseline self-efficacy.(37, 38) The central study app, which directed participants to engage with instruments and prompted them to seek MIH visits for acute illness, and the collaborative efforts of the clinical and study teams may have contributed to the overall cohesiveness of study activities.

It is noteworthy that, although subjects often used MIH evaluations, these encounters were typically not linked to biometric alerts triggered by the study, indicating that other factors prompted these evaluations. Additionally, only about 12% of the app-triggered alerts were associated with an ambulatory encounter within 72 hours, again suggesting that the biometric alerts were not associated with symptom deterioration severe enough to warrant urgent medical evaluation. Further, the acute symptoms that did prompt MIH engagement were not detected by the study app; further intervention refinement is needed to understand the lack of association between these two intervention components.

Despite the lack of coupling of the biometric alerts with healthcare utilization, we still observed a significant use of the MIH service for acute symptom management among participants, which may have been supported by the study app’s ability to connect participants with MIH services as well as encouragement from the virtual pulmonary coaches to summon MIH, thus increasing awareness and ease-of-connection with this service. Using the study app to decrease the cognitive burden on patients when seeking care and allowing them to be cared for in the home further decreased barriers to acute care traditionally experienced by patients. All MIH encounters occurred for participants with the two highest baseline activation score categories, possibly indicating that participants with lower self-efficacy related to their COPD were less likely to proactively engage with a new care delivery service like MIH or didn’t understand its role in their care. Future iterations of the intervention might provide a more hands-on orientation to the MIH component such as a scheduled non-acute home visit or have MIH staff proactively reach out to patients for whom there is a concern for clinical deterioration either due to reported symptoms, biometric markers, or contact with their clinical team.

The intervention also demonstrated signals towards decreased CAT, NIH PROMIS, and mmRC scores, indicating overall COPD symptoms severity after being enrolled in the program, demonstrating preliminary evidence that the intervention may decrease distress related to COPD and improve patient quality of life. This phenomenon is likely multifactorial. Participants may have enjoyed an increased sense of agency and self-efficacy from the pulmonary coaching services, more confidence in their ability to access care when needed, and/or more confidence that COPD symptoms were being adequately supervised. While there may have been concern that increased monitoring would increase anxiety around COPD symptoms or hyperawareness of changes in biometric markers, the monitoring and access to care in this study appeared to improve patient quality of life.

This pilot study had several limitations including its small sample size and nonrandomized design which limits the rigor of outcome measurements. Due to the limitations of the study app and the wearable technology used in this study, enrollment was limited to English-speaking subjects already in possession of smartphones which may have impacted the makeup and representativeness of the sample, particularly because COPD outcomes are impacted by disparities in healthcare delivery.(6, 39) Furthermore, much of the study recruitment was done with electronic communications and relied on subjects to interface with an electronic platform to enroll. The recruited sample is more likely to be comfortable with digital technology and may have exhibited higher fidelity to the digital components of the study when compared to the general population with COPD. During the study, participants were prompted to complete numerous instruments; while this could have provided added information such as changes in subscales, it may have contributed to participant fatigue and decreased the quality of response. It is possible that participants had healthcare encounters outside of the affiliate health system that the study team could not track, potentially limiting the accuracy of the study’s healthcare utilization data.

Further research should be directed at optimizing the cohesiveness of this intervention in preparation for larger effectiveness studies and decreasing the cognitive burden on participants in anticipation of improving the intervention’s generalizability. For example, if clinical information from participants were displayed to ambulatory and MIH clinicians in a way that facilitated proactive clinical response to signals of worsening symptom severity, this may promote even more timely mobile intervention by eliminating the need for patients to self-refer to acute care. This strategy would be further strengthened by the development of algorithms that integrate biometric, patient-reported, and EHR data to increase the accuracy of models that predict acute COPD exacerbation. Finally, as digital medicine continues to emerge as a potential solution to complex problems with healthcare delivery, continued assessment of their implementation and sustainability -- including patient and clinician adoption and continuous use, payor reimbursement, and reach to their target communities -- must be evaluated to ensure successful intervention delivery.

In summary, a multi-component intervention aimed at providing home monitoring and treatment for patients living with COPD leveraging complementary mobile and digital tools was found to be feasible and decreased COPD-related patient distress, demonstrating early promise for improving patient outcomes and enhancing the management of this chronic respiratory condition. Further research is needed to optimize intervention delivery, validate the effectiveness of this strategy, and evaluate its scalability and sustainability in communities.

## Supplementary Material

Supplement 1

## Figures and Tables

**Table 1:** Healthy at Home Intervention Components

Intervention Component	Description	Interaction with other components	All Participants or Opt-In
MyDataHelps App (main study smartphone app)	Houses all participant-facing forms and questionnaires including screening and consent. Prompts participants to complete scheduled assessments. Receives information from biosensors and is linked to participants’ EHR and claims data to aggregate all data streams. Generates momentary assessments triggered by participant responses or biometric data.	All participant-level MyDataHelps data is visible to the tele-pulmonary-rehabilitation and MIH team on the study dashboard. Clinical data can trigger alert to participants through the app suggesting a home assessment from the MIH team and connect participants directly with the MIH visit request-line.	All
Fitbit Smartwatch (and Fitbit app)	Collects biometric data. All data is visible to participants, study team, MIH clinicians and tele-pulmonary-rehabilitation coaches through the MyDataHelps.	Biometric patterns trigger alert to patients suggesting MIH visits. Biometric data is visible to clinical and research teams.	All
Mobile Integrated Health Program (community paramedics and MIH physicians)	Community paramedics are available on-demand to perform home visits to evaluate and treat participants experiencing acute symptoms with support from a supervising physician. The program is specifically equipped to initiate treatment for COPD exacerbation.	MIH team can view participant level data in MyDataHelps. MIH visits offered to participants reporting worsening symptoms or exhibiting concerning biometric data through the study app. Research and tele-pulmonary-rehabilitation team refer participants to MIH team for all acute clinical concerns.	All
Wellinks Virtual Pulmonary Rehabilitation Program	Coaching program to support participant education, treatment-adherence, and goal setting, as well as a virtual pulmonary rehabilitation. Hosted on a separate program-specific app.	Tele-pulmonary-rehabilitation team can view participant level data in MyDataHelps to support coaching plans. tele-pulmonary-rehabilitation team can contact MIH team with any acute concerns requiring clinical evaluation.	Opt-In
Wellinks equipment kit (spirometer, pulse oximeter, exercise equipment	Used with the tele-pulmonary-rehabilitation team to support pulmonary coaching plan and collect additional relevant biometric data.	Spirometry and pulse oximetry data is integrated with the MyDataHelps app and is visible to the study and clinical teams.	Opt-In

**Table 2:** Enrolled Sample Characteristics (N=100)

	All*	Patient Activation Measure 0–55	Patient Activation Measure 56–67	Patient Activation Measure 68–100
**N**	100	9	30	51
**Age**				
Mean (SD)	66 (12)	72 (11)	66 (11)	67 (11)
**Sex n (%)**				
Male	48 (48)	6 (67)	15 (52)	23 (46)
Female	52(52)	3 (13)	14 (48)	27 (54)
**Race n (%)**				
Black or African American	3 (3.0)	0 (0)	0 (0)	2 (3.9)
American Indian or Alaska Native	1(1)	0 (0)	1 (3.3)	0 (0)
Other	6 (6.0)	0 (0)	1 (3.3)	4 (7.8)
White	83 (83.0)	9 (100.0)	26 (92.6)	42 (82.4)
**Ethnicity**				
Hispanic or Latino	15(15.))	0 (0)	2 (7.4)	2 (4.3)
Not Hispanic or Latino (%)	85(85.0)	9 (100)	25 (92.6)	45 (95.7)
**Charleston Comorbidities index (unweighted)**				
Mean (SD)	2.53 (1.81)	2.33 (1.66)	2.52 (1.70)	2.48 (1.76)

* Only 90 participants completed baseline Patient Activation Measure Score

**Table 3:** Intervention Adherence

	All Subjects (N=100)	Patient Activation Measure 0–55 (n=9)	Patient Activation Measure 56–67 (n=30)	Patient Activation Measure 68–100 (n=51)
**Fitbit Use**				
Median Daily Hours worn (IQR)	18.9 (3.8)	18.8 (5.8)	18.9 (4.2)	18.9 (3.1)
Median Days worn (IQR)	114 (183.6)	179 (47)	81 (213)	105 (190)
Median Daily Minutes of heartrate detection	1134 (230)	1127 (349)	1135 (251)	1135 (187)
**Completed Responses to Study Instruments**				
***Scheduled* (n,%)**				
Baseline Demographics (month 0)	93 (93)	9 (100)	31 (100)	51 (100)
COPD Assessment Test	91 (91)	9 (100)	31(100)	51 (100)
NIH-PROMIS COPD	90 (90)	9 (100)	31(100)	51(100)
Patient Activation Measure	91(91)	9 (100)	31(100)	51(100)
mmRC (Modified Medical Research Council) Dyspnea Scale	90 (90)	9 (100)	31(100)	51(100)
Patient Satisfaction (baseline)	87 (87)	9 (100)	29 (93.5)	50 (98.0)
Patient Satisfaction (3 and 6 month)	78 (78)	9 (100)	25 (80.1)	47 (92.2)
Single-item wellness	93 (93)	9 (100)	29 (93.5)	51(100)
***Ad Hoc***				
***CLEAR Sx***				
Patients with at least 1 complete (n, %)	85 (85)	9 (100)	27 (87.1)	47 (92.2)
Total CLEAR Sx completed (mean per patient)	485 (4.85)	102 (11.3)	144 (4.6)	249 (4.9)
**Tele-Pulmonary-rehabilitation Engagement**				
Opted In	55 (55)	8(88.8)	16 (51.6)	31 (60.8)
No opt in	45 (45)	1 (11.1)	14 (45.2)	20 (39.2)
**Healthcare Utilization**				
Any ambulatory encounter within 72 hours of CLEAR Sx	53	6	20	25
MIH Encounters within 72 hours of CLEAR Sx	3	0	0	3
MIH Encounters independent of CLEAR Sx	67	0	24	43
ED visit with no preceding CLEAR Sx Survey	1	0	0	1

**Table 4:** Participant Centered Outcomes

	Timing			
	Baseline	3 Months	6 Months	p
**CAT Score** Mean (SD)	15.96 (7.73)	14.77 (7.79)	13.48 (7.21)	0.017
**NIH PROMIS Score (T score)**				
Physical Limits Mean (SD)	46.60 (8.60	45.25 (7.62)	45.32 (8.96)	0.53
Fatigue Mean (SD)	53.08 (10.09)	51.25 (9.73)	50.63 (10.16)	0.32
**Patient Activation Measure** Mean (SD)	71.68 (14.00)	74.32 (14.31)	77.94 (13.44)	0.04
**Modified Medical Research Council Dyspnea Scale (mmRC)** Mean (SD)	1.18 (1.05)	1.14 (1.04)	1.04 (0.98)	0.76
**Patient Activation Measure 0–55**				
**CAT Score** Mean (SD)	17.22 (6.02)	14.43 (3.99)	15.67 (5.13)	0.573
**NIH PROMIS Score (T score)**				
Physical Limits Mean (SD)	44.67 (3.62)	44.00 (4.64)	48.87 (7.39)	0.226
Fatigue Mean (SD)	57.23 (8.64)	49.89 (7.26)	58.85 (9.92)	0.152
**Patient Activation Measure** Mean (SD)	45.58 (9.31)	56.78 (14.39)	60.00 (17.54)	0.121
**Modified Medical Research Council Dyspnea Scale (mmRC)** Mean (SD)	1.12 (1.25)	1.00 (1.41)	0.80 (1.30)	0.912
**Patient Activation Measure 56–67**				
**CAT Score** Mean (SD)	17.77 (7.19)	16.79 (7.74)	14.67 (7.08)	0.470
**NIH PROMIS Score (T score)**				
Physical Limits Mean (SD)	48.88 (8.30)	47.10 (5.91)	47.22 (7.19)	0.646
Fatigue Mean (SD)	55.90 (7.77)	54.36 (8.34)	52.72 (8.12)	0.478
**Patient Activation Measure** Mean (SD)	63.93 (2.93)	68.13 (9.65)	73.57 (8.46)	<0.001
**Modified Medical Research Council Dyspnea Scale (mmRC)** Mean (SD)	1.23 (0.90)	1.43 (0.98)	1.25 (0.62)	0.722
**Patient Activation Measure 68–100**				
**CAT Score** Mean (SD)	14.67 (8.16)	13.85 (8.27)	12.68 (7.59)	0.537
**NIH PROMIS Score (T score)**				
Physical Limits Mean (SD)	45.56 (9.15)	44.54 (8.66)	43.78 (9.76)	0.685
Fatigue Mean (SD)	50.77 (10.98)	49.93 (10.52)	48.08 (10.20)	0.547
**Patient Activation Measure** Mean (SD)	80.84 (9.25)	80.34 (12.63)	82.82 (11.49)	0.623
**Modified Medical Research Council DyspneaScale (mmRC)** Mean (SD)	1.16 (1.12)	1.02 (1.00)	1.00 (1.05)	0.761

## Data Availability

The protocols and datasets used during the current study are available from the corresponding author upon reasonable request.
