# Supplementary material for: Healthy at Home for COPD: An Integrated Digital Monitoring, Treatment, and Pulmonary Rehabilitation Intervention
Source: Res Sq. 2024 Nov 15:rs.3.rs-5084150. Preprint. [Version 1] doi: 10.21203/rs.3.rs-5084150/v1 (PMC11601823; doi:10.21203/rs.3.rs-5084150/v1)
Supplement: Supplement 1 [file NIHPPRS5084150V1-supplement-1.pdf]

Supplementary Files

This is a list of supplementary files associated with this preprint. Click to download.

- HealthyatHomeSupplementalTable1.docx
- HealthyatHomeSupplementalTable2.docx
